# Effectiveness of emergency obstetric care training at the regional level in Ukraine: a non-randomized controlled trial

**DOI:** 10.1186/s12884-022-04458-9

**Published:** 2022-02-22

**Authors:** Iryna Mogilevkina, Vitaliy Gurianov, Gunilla Lindmark

**Affiliations:** 1 Institute of Postgraduate Education, Bogomolets National Medical University, Schevchenko Av. 13, 01601 Kyiv, Ukraine; 2 Donetsk National Medical University, 84331 Kramatorsk, Donetsk Oblast, UA Ukraine; 3 Health Management Department, Bogomolets National Medical University, 01601 Kyiv, Ukraine; 4 Department of Women´s and Children´s Health, Uppsala University, 75105 Uppsala, SE Sweden

**Keywords:** Emergency obstetric care, Training, Evaluation, Effectiveness, Health outcomes, Postpartum hemorrhage, Vacuum extraction, Ukraine

## Abstract

**Background:**

Emergency obstetric care training, using Advances in Labour and Risk Management (ALARM) International Program (AIP) was implemented in Ukraine, a country with universal access to skilled perinatal and obstetric care but restricted resources. A total of 577 providers (65.5% of total) from 28 maternal clinics attended a 5-day training session focused on the five main causes of maternal mortality, with hands-on skill workshops, pre- and post- tests, and an objective structured clinical examination. The effects of this emergency obstetric care training on maternal outcomes is the subject of this paper.

**Methods:**

A non-randomized controlled trial was conducted. The pilot areas where the training was implemented consisted of 64 maternity clinics of which 28 were considered as cases and 36 non-participating clinics were the referents. Data on maternal outcomes were collected for a 2-year span (2004-2005) prior to the trainings, which took place 2006-2007 and again after implementation of the trainings, from 2008 to 2009. Information was collected from 189,852 deliveries. Outcomes for the study were incidences of operative delivery and postpartum hemorrhage. Non-parametric statistics, meta-analyses, and difference in difference (DID) estimation were used to assess the effect of the AIP on maternal indices.

**Results:**

DID analysis showed that after the training, compared to the referents, the cases had significant reduction of blood transfusions (OR: 0.56; 95%CI: 0.48-0.65), plasma transfusions (OR: 0.70; 95%CI: 0.63-0.78), and uterus explorations (OR: 0.64; 95%CI: 0.59-0.69). We observed a non-significant reduction of postpartum hemorrhage ≥1000 ml (OR: 0.92; 95%CI: 0.81-1.04; *P* = 0.103). Utilization of vacuum extraction for vaginal delivery increased (OR: 2.86; 95%CI: 1.80-4.57), as well as forceps assisted delivery (OR: 1.80; 95%CI: 1.00-3.25) and cesarean section (OR: 1.11; 95%CI: 1.06-1.17). There was no change in the occurrence of postpartum hysterectomy and maternal mortality.

**Conclusions:**

After one week of Emergency Obstetrics Care training of the obstetric staff in a setting with universal access to perinatal and obstetric care but restricted resources, an association with the reduction of postpartum hemorrhage related interventions was observed. The effects on the use of vacuum extraction and cesarean section were minimal.

**Trial registration:**

Retrospectively registered 071212007807 from 07/12/2012.

## Background

Training programs in Emergency Obstetrics Care (EmOC) are common, and the results of such trainings are well described [1–7]. There are four levels to assess effectiveness of training in EmOC: first, the reaction of participants, second, the improvement of knowledge, and skills, third, changes in providers’ behavior and practices, and fourthly, EmOC availability and health outcomes [8]. That said, detailed analyses of the effects of a training program on outcome measures, including maternal and perinatal mortality and morbidity, were for the most part, contradictory. Whereas some studies demonstrate improvement in perinatal outcomes [9] and reduction in postpartum hemorrhage (PPH) [10], others show no effect of obstetric skills training on the rate of red blood transfusion for PPH [11], the rate of PPH [12], or the adverse outcome index [13].

The Society of Obstetricians and Gynecologists of Canada established the Advances in Labour and Risk Management (ALARM) International Program (known collectively as AIP) in 1998 to assist in the global reduction of maternal and neonatal mortality and morbidity. AIP was designed to motivate and encourage health professionals to improve the delivery of emergency obstetric care in low and middle income countries [14]. The 5-day AIP course is based on adult learning principles that promote a collaborative, multidisciplinary approach of working and learning together. It focuses on the five main causes of maternal mortality: obstructed labor, hemorrhage, sepsis, hypertensive disorders and complications due to unsafe abortion [14]. Participants are also sensitized to factors that impede women’s access to reproductive health services and information [15].

Despite universal access to perinatal and obstetric care, health outcomes for mothers and infants in Eastern European countries are far less favorable than those reported from Western Europe. In Ukraine, despite nearly all women deliver in hospital, attended by an obstetrician-gynecologist, maternal mortality is still much higher than in Western Europe [16].

From 2006 to 2009, Ukraine was one of eleven project countries within the International Federation of Gynecology and Obstetrics (FIGO), “Saving Mothers and Newborns Initiative”, a program that aimed to contribute to the reduction of maternal and newborn morbidity and mortality in targeted countries. The primary tool to achieve those objectives in Ukraine was the AIP [17].

The AIP program in Ukraine was implemented in two pilot regions, Donetsk and Vinnytsia with Ministry of Health (MOH) support. We hypothesized that AIP training would be followed by a decline in important adverse maternal and perinatal outcomes. The aim of this publication is to evaluate the effect of the AIP on selected maternal outcomes at the pilot facilities.

## Methods

**Fig. 1:**
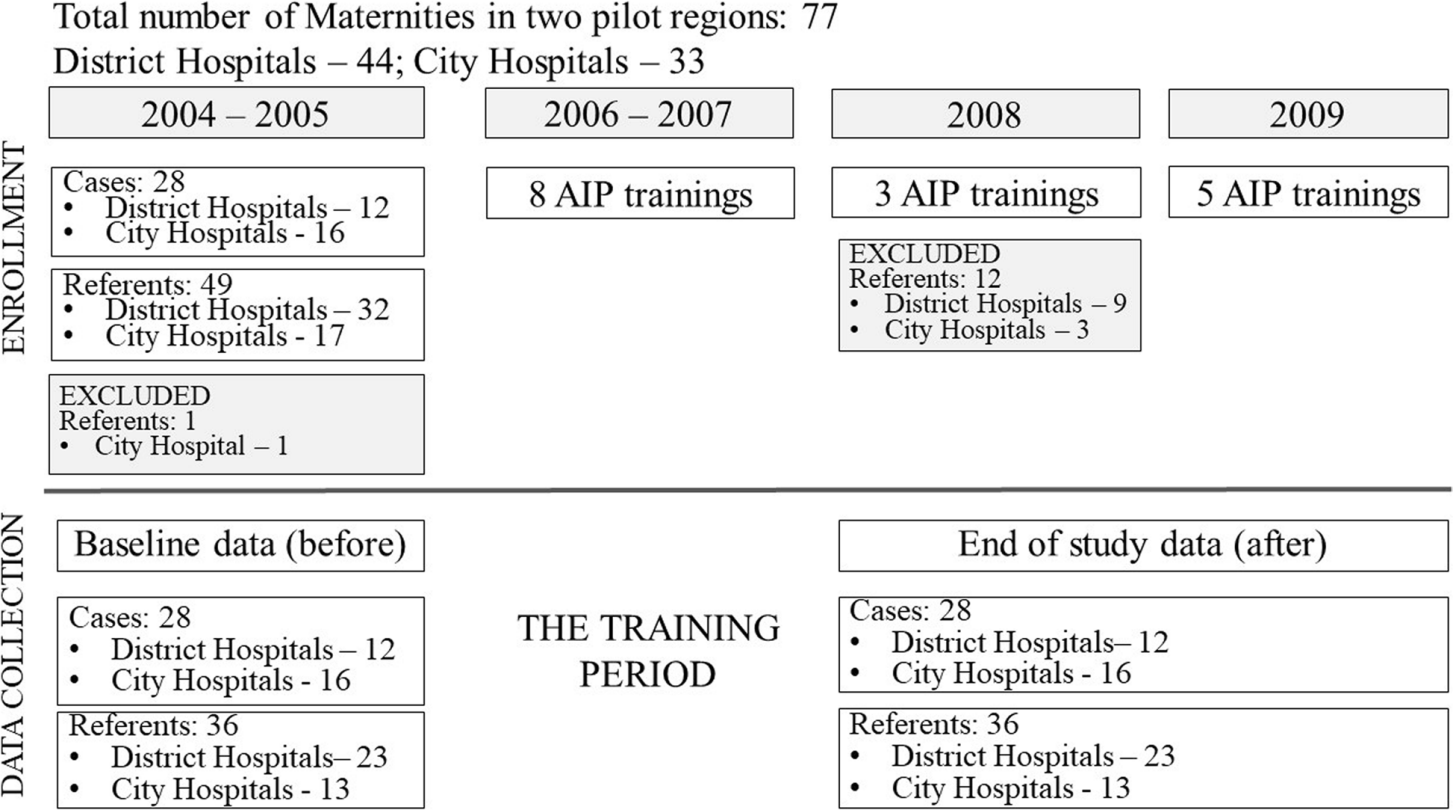
Study design to evaluate effectiveness of the emergency obstetric care training in Ukraine Obstetrical facilities from the pilot area (Donetsk and Vinnytsia regions) included in the ALARM International trainings were considered as cases whereas those that did not participate were the referents

To study the effect of the AIP on maternal and neonatal outcomes, a quasi-experimental design was used. A non-randomized controlled trial was conducted (Fig. 1).

In 2005, the Donetsk region, a highly industrial region in the eastern part of Ukraine, was home to approximately 1.2 million women of reproductive age. The number of deliveries that year was 35,319, cared for in 44 in-patient Maternal Care facilities (17 district hospitals and 27 city hospitals). These facilities were staffed with approximately 1600 midwives and 1300 obstetrician-gynecologists. The second pilot region was Vinnytsia, a rural region in central Ukraine with around 400,000 women of reproductive age. Here, the number of deliveries in 2005 was 14,640, in 33 in-patient Maternal Care facilities (27 district hospitals and 6 city hospitals) staffed by approximately 700 midwives and 400 obstetrician-gynecologists. Approximately half of the providers in both regions were involved in the care of women and neonates during delivery and labor.

Fourteen of 44 facilities in Donetsk and 14 of 33 facilities in Vinnytsia region were designated as project clinics with a total of 881 targeted providers. Those facilities were selected by Regional Public Health Administrations (RPHAs). The 28 facilities from the pilot area included in the AIP training initiative were considered as cases, whereby the remaining 49 facilities served as referents. One city hospital was excluded from the referent group as it was a site used for another project. Each of the 76 facilities was considered as a unit.

During 2006-2007, a total of 28 pilot facilities in the two Ukrainian regions participated in eight AIP training sessions. In 2008, an additional three trainings were conducted. On the request of RPHAs, professionals from 12 referent maternity units were invited to the trainings. Therefore, those 12 units were excluded from the referents. Five AIP training sessions were arranged in 2009. Again, on the request of the RPHAs, an additional 11 facilities from the referent group were invited to the AIP in the second half of 2009. Since those facilities were involved so late in the trainings, no effect on maternal and perinatal outcomes was expected in 2009. Therefore, we decided not to exclude them from the referents. Distribution of facilities by annual number of deliveries is presented in Table 1.

**Table 1:** Distribution of facilities involved in the study by number of deliveries per year in 2004

Number of deliveries per year	Cases, n	Referents, n	Total, n
< 500	11	21	32
500 - 999	7	12	19
1000 - 2000	9	3	12
> 2000	1	-	1
Total	28	36	64

In total, 342 obstetrician-gynecologists and 235 midwives from the case clinics were trained during 2006-2009 and average coverage for the staff training was 65.5%, varying from 28.6 to 100%. Staff retention rate was close to 100% in all maternity units in the area, during the period under observation.

The AIP training educational environment includes interactive plenary sessions and hands-on skill workshops with obstetrical mannequins, thus allowing all participants the opportunity to address individual learning needs. Sessions were accompanied by pre- and post- tests which measured knowledge and practice skills, including objective structured clinical examinations that required the participant to demonstrate clinical skills [14, 15]. A 2-hour monitoring and refresh session on PPH was conducted in the case units in late 2009.

In Ukraine, statistics are based upon annual reports from the regional departments of medical statistics, which rely upon quarterly hospital reports. No case-based registers were available in the country at the time of the AIP implementation. Hospital reports compiled information from local case records and contained some aggregated data but no personal characteristics of the patients. Before the project was started, a specially designed hospital record form was developed and approved by the RPHAs for collecting hospital data in the suggested project area. Data cannot be linked to any individual patient. These forms were completed by the statisticians in the maternity units from official hospital statistics, since 2001 retrospectively, and since 2005 prospectively. They were checked for consistency by chief-doctors, collected and entered into the database by the investigator.

Information was collected on an annual basis for number of deliveries, live-births, percentage of pre-term births, multiple births, vacuum and forceps delivery, cesarean section (CS), PPH, cases where exploration of uterus was performed, blood or plasma transfusion, postpartum hysterectomy along with Maternal Mortality Ratio per 100,000 live-births (MMR), perinatal deaths (per 1000), and neonatal morbidity (per 1000). PPH was recorded for blood loss 1000 ml or more within 24 h of the birth process, regardless of the route of delivery, and was evaluated by weighing the amount/output [18, 19], according to Ukraine MOH recommendations. To more accurately determine the actual postpartum blood loss and to diminish the effect of amniotic fluid and urine (in vaginal birth) on PPH quantification, after delivery of the placenta, large absorbent pads were changed in vaginal birth and amount of fluid in suction canister was counted for in cesarean section. Irrigation fluid was collected in another canister. All blood-soaked materials and clots as well as fluid volume collected were weighed on a scale in grams. The dry item’s gram weight was subtracted. 1gram weight was considered equal to 1 milliliter of blood loss volume [20]. Total PPH blood loss was recorded after the obstetric team judged that PPH had stopped. Information on 189,852 deliveries was collected.

For this study, data were selected from the database for a 2-year span (2004-2005) prior to trainings and then the 2 years (2008-2009) following the training period at all maternity units of the project area, including both cases and referents. These 2 interim years, 2006-2007 were roll-out periods, defined as transition periods and therefore data for that period were not included in the study. Data for two referent maternity units were collected only for one-year after (2008), since due to the facility closing for renovations 2009 data were not available.

Data obtained for each unit and each year were treated separately as continuous relative indices. As samples for all indices were not normally distributed (Shapiro-Wilk W test), the median with interquartile range (IQR) was calculated prior to and after the training (before, after) in two groups (the cases and the referents). In the univariate analyses, Wilcoxon rank-sum tests were applied. The JMP (SAS Institute, Cary, NC, 1994) program package was used for data-entering and analyses.

Taking into account the large difference in the number of births at the facilities and non-normal distribution of samples, in order to provide a numerical estimate of the overall effect of interest, a meta-analysis was done to calculate the weighted summary proportions under the fixed (Freeman-Tukey transformation (arcsine square root transformation) [21] or random [22] effects model (after test for heterogeneity). A meta-analysis integrates the quantitative findings of maternal indices from each unit and each year for the cases and the referents, the groups before and after the AIP implementation. MedCalc software 20.013 was used to calculate the weighted average indices with 95% CI [23].

Difference-in-differences (DID) analysis was applied to compare outcome changes overtime between the case and the referent groups to evaluate the impact of the AIP on maternal indices by linear regression model [24]. Statistical software EZR v.1.54 (graphical user interface for R statistical software version 4.0.3, R Foundation for Statistical Computing, Vienna, Austria) [25] was used to calculate DID estimators with 95%CI and OR of DID with 95%CI.

 The study was approved by the Ethical Committee of the Institute of Medical Problems of Family of the Donetsk National Medical University (DNMU) (Protocol 24, November 7, 2012). The study was retrospectively registered at the DNMU Register, Nu 071212007807 from 07/12/2012.

## Results

During the study period there was an increase in the number of births both in the case maternity units (from 47,838 in 2004-2005 to 61,116 in 2008-2009) and in the referents (from 38,032 in 2004-2005 to 42,866 in 2008-2009) with more pronounced increase among the cases.

Non-parametric statistics (Table 2) revealed that at the baseline CS and forceps assisted delivery was more often utilized among the cases. There were no differences in other indices.

**Table 2:** Maternal outcomes and some obstetric interventions before and after the training period

Nu	Indices	Before (2004 – 2005)						After (2008 – 2009)						Before vs After			
		Cases (n=28)		Referents (n=36)		Z_***1-2***_	***P***_***1-2***_	Cases (n=28)		Referents (n=36)		Z_***3-4***_	***P***_***3-4***_	Cases (n=28)		Referents (n=36)	
		Median	IQR	Median	IQR			Median	IQR	Median	IQR						
		1		2				3		4				Z_***1-3***_	***P***_***1-3***_	Z_***2-4***_	***P***_***2-4***_
	Number of deliveries	47 838		38 032				61 116		42 866							
1	Vacuum assisted delivery, %	0	0-0 Max 0.77	0	0-0 Max 1.71	0.43	0.669	0.31	0-0.98 Max 4.58	0	0-0.44 Max 2.53	3.59	**0.000**	-5.64	**< 0.000**	2.51	**0.012**
2	Forceps assisted delivery, %	0.04	0-0.30 Max 2.24	0	0-0 Max 1.17	2.95	**0.003**	0	0-0 Max 1.40	0	0-0 Max 0.70	1.56	0.118	3.10	**0.002**	-1.85	0.064
3	Cesarean section, %	11.30	8.82-13.09	8.33	5.79-13.05	2.42	**0.015**	12.82	9.70-15.59	10.54	7.42 -14.47	2.50	**0.012**	-1.93	0.053	1.959	0.050
4	Postpartum hemorrhage, %	2.24	1.22-5.98	1.83	1.19-4.11	1.19	0.233	1.98	0.85-2.70	1.72	1.01-2.75	0.07	0.941	2.19	**0.029**	0.998	0.318
5	Uterus exploration, %	5.49	1.58-8.88	6.12	3.16-11.89	-1.73	0.084	2.98	1.32-4.94	4.44	2.71-8.09	-3.25	**0.001**	2.46	**0.014**	-1.94	0.053
6	Blood transfusion, %	1.28	0.61-2.20	1.70	0.69-2.49	-0.84	0.399	0.84	0.36-1.36	0.92	0.31-1.71	-0.63	0.528	2.58	**0.010**	-2.88	**0.004**
7	Plasma transfusion, %	2.18	0.57-4.36	1.93	1.39-5.90	-0.96	0.337	2.05	0.70- 3.53	1.80	0.88-4.30	-0.41	0.684	0.32	0.746	-1.23	0.219
8	Hysterectomy postpartum, %	0.11	0-0.25 Max 2.04	0	0-0.34 Max 2.17	0.003	0.998	0.15	0-0.31 Max 1.74	0.18	0-0.39 Max 1.09	-0.66	0.506	-0.62	0.532	0.97	0.333

The median with interquartile range (IQR) is presented. Maximum is added where medians and IQRs were equal to 0. Wilcoxon rank-sum test was used to study difference between the cases and the referents before and after the training period. *P*-values<0.05 were considered significant (seen in bold). Data on Maternal Mortality Ratio and Maternal Mortality Ratio related to postpartum hemorrhage was excluded from the Table as medians and IQRs for all points were equal to 0

After the training there was a minor increase in vacuum assisted delivery in both groups. Use of forceps assisted delivery declined in both groups, but only for the cases was that decrease significant. CS rate increased from 2004 to 2005 to 2008-2009, yet it was not significant for either the cases or the referents.

PPH with blood loss 1000 ml or more and uterus exploration decreased significantly among the cases, whereas PPH decline among the referents was not significant, neither for the rate of uterus exploration. Blood transfusions were reduced significantly in both groups, but no change in plasma transfusion and postpartum hysterectomy was found after the training, not for the cases nor the referents. No changes in MMR nor MMR related to PPH was observed.

At the end of the study (2008-2009), vacuum assisted delivery was slightly more frequent among the cases. CS rate was still higher among those units involved in the AIP, whereas uterus exploration was less used as compared to the referents.

When meta-analyses were performed, there was no difference between the cases and the referents at the baseline.

**Fig. 2:**
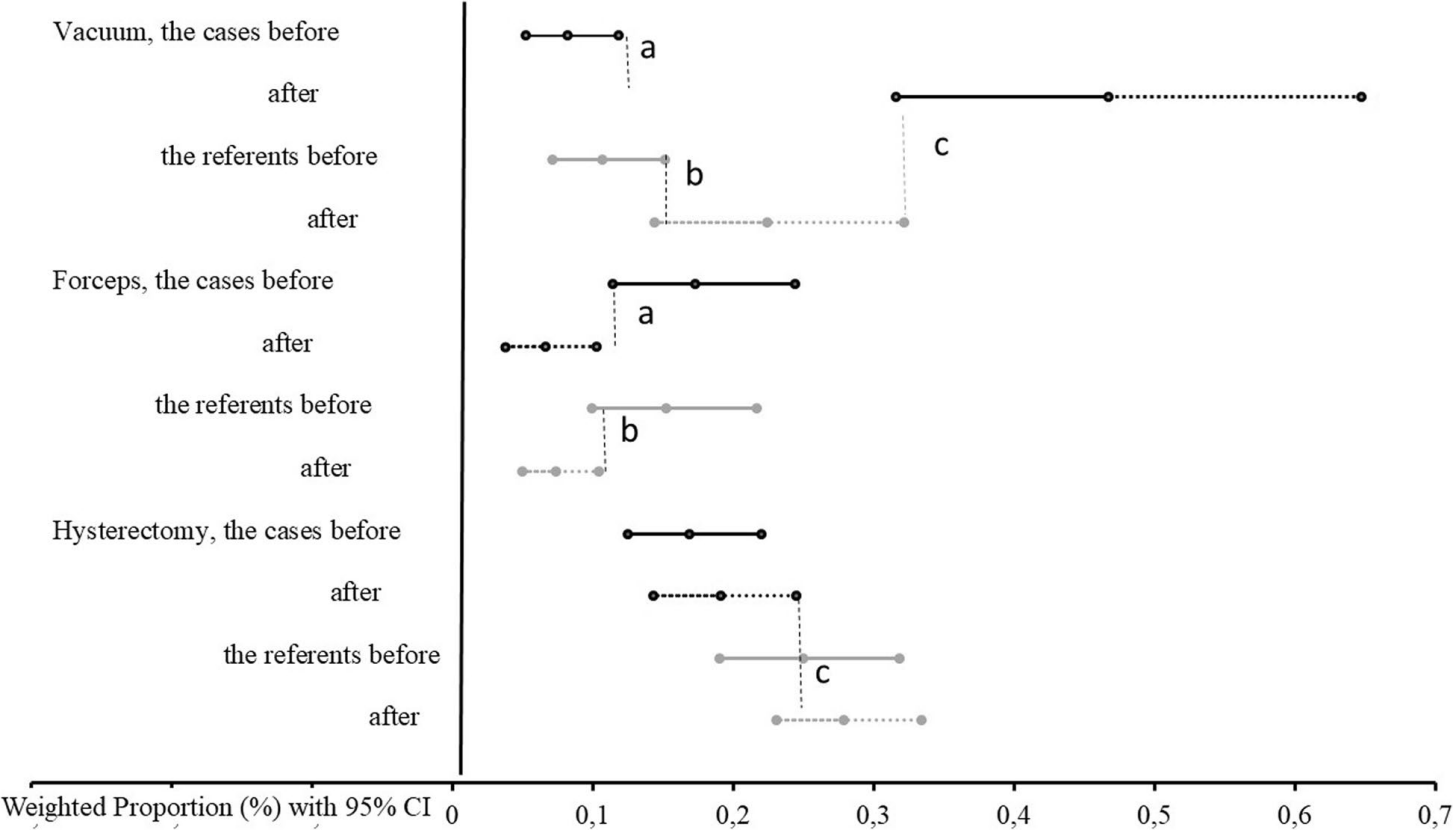
Effect of the emergency obstetric care training on instrumental vaginal delivery and postpartum hysterectomy rate Weighted average indices calculated using meta-analyses framework for the cases (n=28) and the referents (n=36) before and after the training with 95%CIs (the whiskers). Black lines represent indices’ values for the cases and grey lines - for the referents. Solid lines indicate groups “before”, dotted lines – “after” the training. *P*<0.05 for the difference of ^a^ the cases before compared with the cases after, ^b^ the referents before and after, ^c^ the cases and the referents after the training. CI: confidence interval

**Fig. 3:**
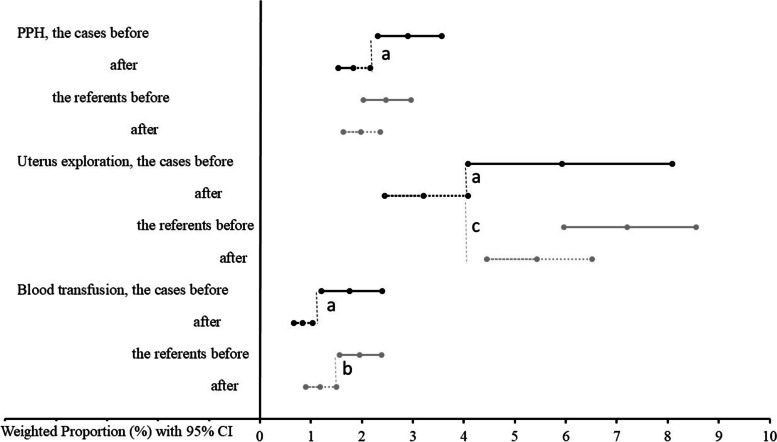
Weighted average rates of postpartum hemorrhage and related activities on regional scale after staff training Weighted average indices calculated using meta-analyses framework for the cases (n=28) and the referents (n=36), before and after the training with 95%CIs (the whiskers). Black lines represent indices’ values for the cases and grey lines - for the referents. Solid lines indicate groups “before”, dotted lines – “after” the training. *P*<0.05 for the difference of ^a^ the cases before compared with the cases after, ^b^ the referents before and after, ^c^ the cases and the referents after the training. CI: confidence interval

Data on weighted average indices with 95% CI demonstrated significant changes and are presented in Figs. 2 and 3. Among the cases, after the AIP implementation there was a significant increase in vacuum-assisted delivery, and decreases noted in the following: forceps assisted delivery, PPH, uterus exploration and blood transfusion. Also at the referent maternity units, there was a significant increase in vacuum assisted deliveries, a decrease in forceps assisted delivery and in blood transfusions.

At the end of the study (2008-2009) vacuum assisted deliveries occurred 2.1 times more often among the cases, whereas uterus exploration was 2.2 times more often and postpartum hysterectomy was 1.5 times more common among the referents.

There were no significant changes in CS rate between the case (11.3; 95% CI: 10.1-12.5 vs. 13.2; 95% CI: 11.7-14.8) and referent maternity units (9.7; 95% CI: 8.3-11.2 vs. 11.2; 95% CI: 9.9-12.7) during the study period.

There were 12 maternal deaths at the baseline and 18 maternal deaths at the end of the study.

Weighted indices showed no difference in MMR per 100,000 live births between the cases and the referents neither before (35.8; 95% CI: 21.0-57.2 vs. 52.2; 95% CI: 31.8-80.7) nor after the training period (37.0; 95% CI: 23.4-55.7 vs. 45.1; 95% CI: 27.3-70.1). The same was true for MMR related to PPH: 28.8; 95% CI: 15.7-48.4 vs. 45.8; 95% CI: 26.9-72.9, and 28.8; 95% CI: 12.5-38.3 vs. 37.1; 95% CI: 21.2-60.3.

Table 3 summarizes the DID analysis’s outcomes.

**Table 3:** Effect of the AIP training on maternal indices: difference in difference (DID) estimation

Outcome variable	Referents, N (%)		Cases, N (%)		DID		p
	Before, n=38,032	After, n=42,866	Before, n=47,838	After, n=61,116	DID estimator (95%CI)	OR (95%CI)	
Postpartum hemorrhage	905 (2.38)	747 (1.74)	1255 (2.62)	1081 (1.77)	-0.22 (-0.48 to 0.04)	0.92 (0.81-1.04)	0.103
Uterus exploration	2929 (7.70)	2521 (5.88)	3977 (8.31)	2530 (4.14)	-2.35 (-2.80 to -1.91)	0.64 (0.59-0.69)	<0.001
Blood transfusion	851 (2.24)	635 (1.48)	1008 (2.11)	476 (0.48)	-0.57 (-0.80 to -0.35)	0.56 (0.48-0.65)	<0.001
Plasma transfusion	1415 (3.72)	1120 (2.61)	2181 (4.56)	1392 (2.28)	-1.12 (-1.50 to -0.85)	0.70 (0.63-0.78)	<0.001
Hysterectomy postpartum	101 (0.27)	109 (0.25)	75 (0.16)	109 (0.18)	0.03 (-0.05 to 0.13)	1.19 (0.80-1.77)	0.439
Vacuum assisted delivery	41 (0.11)	111 (0.26)	48 (0.10)	420 (0.69)	0.44 (0.33 - 0.54)	2.86 (1.80-4.57)	<0.001
Forceps assisted delivery	80 (0.21)	23 (0.05)	80 (0.17)	47 (0.08)	0.07 (0.00 - 0.13)	1.80 (1.00-3.25)	0.041
Cesarean section	4706 (12.37)	5884 (13.73)	6278 (13.12)	9734 (15.93)	1.45 (0.82 - 2.08)	1.11 (1.06-1.17)	<0.001

N in each box of the second row corresponds to number of deliveries in the groups “before” and “after” the AIP training implementation. Difference in difference (DID) estimator was estimated using linear regression model. DID is presented as estimator (%) with 95% CI, and OR with 95%CI. The *P*-value is presented for DID estimator. CI: confidence interval; OR: odds ratio

The estimates in the models for the maternal indices suggest that likelihood of uterus exploration, blood transfusion, and plasma transfusion was less for the cases. Reduction was significant. Cases of PPH were non-significant, less after the AIP training. Vacuum assisted, forceps assisted delivery and CSs were more common after the training. The effect of the AIP training on operative delivery was significant. No effect of the AIP training was found on postpartum hysterectomy, MMR, and MMR related to PPH (Table 4).

**Table 4:** Effect of the AIP training on maternal death: difference in difference (DID) estimation

Outcome variable	Referents, N (per 100,000)		Cases, N (per 100,000)		DID		p
	Before n=38,076	After n=42,872	Before n=47,992	After n=61,330	DID estimator (95%CI)	OR (95%CI)	
Maternal Mortality Ratio	5 (13.1)	5 (11.7)	7 (14.6)	13 (21.2)	8.1 (-14.8 to 31.0)	1.64 (0.35-7.66)	0.490
Maternal Mortality Ratio related to postpartum hemorrhage	2 (5.25)	1 (2.33)	2 (4.17)	3 (4.89)	3.6 (-8.2 to 15.5)	2.64 (0.13-52.8)	0.546

N in each box of the second row corresponds to number of live-births in the groups “before” and “after” the AIP training implementation. N in boxes related to maternal mortality ratio are numbers of women who died. Index is calculated per 100,000 live-births. Difference in difference (DID) estimator was estimated using linear regression model. DID presented as estimator (per 100,000) with 95% CI, and OR with 95%CI. The *P*-value is presented for DID estimator. CI: confidence interval; OR: odds ratio

## Discussion

 Our findings show that implementation of the AIP training at regional level in Ukraine resulted in a significant decline in PPH related interventions, namely uterus exploration (by 36%), blood transfusion (by 44%), and plasma transfusion (by 30%). We observed a non-significant 8% reduction in PPH among the cases. There was a small but significant increase in the use of vacuum (by 186%), forceps assisted delivery (by 80%), and CS (by 11%) after the training. Postpartum hysterectomy, MMR and MMR related to PPH were not affected by the AIP.

This paper represents the first time that the results of the AIP, with emphasis on outcome measures, are described at the regional level in a country with restricted resources but universal access to skilled perinatal and obstetric care.

When the ALARM course was introduced, litigation against those who had taken the course, was shown to decline [26]. Our previous evaluations have reported the experiences of AIP in developing professionals’ responsibility in promoting women’s sexual and reproductive health and rights in Ukraine [27], as well as demonstrated the project’s successes and challenges [17]. A cluster-randomized trial was initiated in Mali and Senegal in 2007 to evaluate effectiveness of complex intervention with AIP training, in reducing maternal mortality [28]. Maternal death review combined with implementation of best practices was effective to reduce hospital base maternal mortality in the first level referral hospitals [29]. The AIP training conducted at a Teaching and Referral Hospital in Kenya with 80% staff coverage demonstrated significant increase in Oxytocin use for active management of the third stage of labor and a decrease in PPH [30].

For more than 30 years, oxytocin has been used routinely after birth in Ukraine to prevent PPH. The data from our training project indicate that AIP training was associated with a downward trend in PPH. Including a quantitative measurement of blood loss during the AIP training could improve the accuracy and reliability of gravimetric technique [31] and may have resulted in a lower threshold for diagnosing PPH [32]. Since there was a significant increase in operative deliveries [32] with increased PPH risk [31] this might lead to less significant PPH reduction after the AIP training. CS increase was significant after the AIP training which is in line with other studies [32]. Vacuum assisted delivery increased in both groups after the training.

Historically, blood transfusion rate is regarded as a more objective and reliable indicator of blood loss [32]. We have found that the AIP training was associated with significant reduction in blood and plasma transfusion. In the referent clinics there was also a significant reduction in blood transfusions. This can probably be explained by the MOH PPH protocol with a strong recommendation to proceed with blood transfusion only if hemoglobin is less than 70 g/l and hematocrit is less than 0.25 l/l (MOH Order 782, 29.12.2005). We can’t exclude effect of that order also in the cases.

Significant reduction in uterus exploration is another indirect measure of PPH decline. If after placenta delivery uterine bleeding does not stop promptly, PPH management protocol is applied [15]. When less invasive measures are not successful (uterotonics, external and bimanual uterus compression) uterus exploration is utilized if doubt exists about the potential for retained products or blood clots.

Our findings may reflect the broad coverage in AIP of many different aspects of PPH prevention / management [33], including partograph use, delayed and obstructed labor management, operative birth, prevention of primary CS, vaginal birth after CS, shoulder dystocia, multiple birth, premature rupture of the membrane, maternal infection, preeclampsia and implementation of the AIP PPH prevention/management evidence-based protocol, which further has been introduced and promoted by FIGO [34]. Therefore, not only training in PPH management as such, but coverage of all risk factors for excessive postpartum bleeding during the training was crucial [15] to improve outcomes. It is not likely that the short PPH monitoring and refresh sessions in late 2009 did effect the results obtained.

The PPH management protocol was introduced in Ukraine by the MOH (Order 676, 31.12.2004), but still there was a difference in the need for PPH related interventions between those who participated in the AIP course and those who did not, clearly related to the AIP training. Items included during the AIP training were: correct active management of the third stage of labor technique with controlled cord traction only with well contracted uterus and after signs of separation of the placenta [34]; uterotonics prescription according to FIGO recommendations; use of external and bimanual uterus compression prior to uterus exploration when examined placenta is complete; aorta compression as an early intervention to stop massive bleeding and reduce blood loss, and intrauterine balloon tamponade with glove to reduce the need for surgical intervention.

Postpartum hysterectomy and maternal death are the most severe PPH related morbidities [35]. Our data showed no changes in postpartum hysterectomy, MMR and MMR related to PPH neither in the maternity units where AIP was implemented nor where it was not, but the sample size was not large enough to demonstrate any change in these rare events. The MMR in the area corresponds to the World Bank estimates of MMR in Ukraine during 2004-2009 [36].

There are many factors related to maternal and perinatal outcomes. Therefore, changes in health outcomes, attributed to training in EmOC, are difficult to measure [8]. During the study period several other activities in the country could affect maternal outcomes. Since 2002, the Maternal and Infant Health Project, an 8-year project implemented by John Snow Inc Research and Training Institute, has worked successfully in the country with the aim to improve the quality of services by implementing evidence-based perinatal technologies [37, 38]. Since 2005 there has been active promotion of the Kiwi Vacuum Assisted Delivery System use in Ukraine. Video with training material from Dr. Aldo Vacca was widely distributed within the country. Still, as demonstrated from our data, vacuum assisted vaginal delivery is uncommon in Ukraine even if it significantly increased after the AIP. This is similar to a study from Mozambique [39].

 An acknowledged weakness of our approach is that the project sites were not randomly selected, and it was not possible to stratify facilities by work load at the regional level since project sites selection was the RPHAs decision.

As the AIP Program was the only new Program introduced in the project facilities (cases) since 2006, it is likely that the changes observed in some maternal indices are associated with the AIP training. The observed differences in the maternal indices are subject for many unobserved confounding factors. As data obtained for analysis were only aggregated data it was impossible to adjust DID PPH estimator by other important factors with known effect on PPH. Collection of personalized information could resolve this issue, but was not the case in Ukraine at the time of the study.

Other limitations of the study are that the data used are relatively old and since the study examined the only the Ukrainian population, the effects in other patient populations is unknown.

The main strength of our study is the use of a large dataset to reflect change at regional level and lack of obstetric employee turnover during the study period. There was a constant increase in number of births in Ukraine and also in the project area during 2004-2009 [40]. The number of births increased more in the case clinics.

The experience from this study of a large-scale but time-limited training intervention of obstetric staff indicates that even a short training can significantly decrease the incidence of interventions related to a potentially life-threatening complication such as PPH and demonstrate a downward trend in PPH. The positive effect also seems to be extended for a longer time period. Among the activities necessary to improve obstetric care and maternal and perinatal health, goal-oriented training of staff should be a priority, especially in settings where skilled staff is the most important resource.

## Conclusions

Re-training of obstetric staff with focus on EmOC in a setting with universal access to perinatal and obstetric care and restricted resources had positive effects as evidenced by a decrease in the incidence of PPH related interventions and downward trend in PPH.

## Data Availability

The datasets used and/or analysed during the current study are available from the corresponding author on request.

## Abbreviations

EmOC: Emergency Obstetrics Care
FIGO: The International Federation of Gynecology and Obstetrics
ALARM: Advances in Labour and Risk Management
AIP: ALARM International Program
PPH: Postpartum Hemorrhage
MMR: Maternal Mortality Ratio
CS: Cesarean Section
MOH: Ministry of Health
RPHAs: Regional Public Health Administrations

## Declarations

With support from the Regional Public Health Administrations data were collected using a specially designed hospital’s record form completed from the official hospital statistics, contained aggregated data but no personal characteristics of the patients. Data cannot be linked to any individual patients. Patients’ informed consent was waived by the Ethical Committee of the Institute of Medical Problems of Family of the Donetsk National Medical University.
